# P01.09. Effect of Maeg-Moon-Dong-Tang on production and secretion of respiratory mucus

**DOI:** 10.1186/1472-6882-12-S1-P9

**Published:** 2012-06-12

**Authors:** C Lee, H Sung, J Kim

**Affiliations:** 1 Chungnam National University School of Medicine, Daejeon, Republic of Korea; 2 Dongguk Univ-Seoul, Graduate School of Oriental Medicine, Seoul, Republic of Korea; 3 Dongguk Univ-Seoul, Graduate School of Oriental Medicine, Seoul, Republic of Korea

## Purpose

Effects of maeg-moon-dong-tang (MMT) on ATP- or TNF-alpha- or PMA- or EGF-induced MUC5AC mucin production and gene expression from human airway epithelial cells and the increase in airway epithelial mucosubstances of rats were investigated.

## Methods

Confluent NCI-H292 cells were pretreated for 30 min in the presence of MMT and treated with ATP (200μM) or PMA (10ng/ml) or EGF (25ng/ml) or TNF-alpha (0.2nM) for 24 hours, to assess the effect of MMT both on ATP- or PMA- or EGF- or TNF-alpha-induced MUC5AC mucin production using enzyme-linked immunosorbent assay (ELISA) and on gene expression by the same inducers using reverse transcription-polymerase chain reaction (RT-PCR). At the same time, hypersecretion of airway mucus was induced by exposure of rats to SO_2_ during 3 weeks. Effect of orally-administered MMT during 2 weeks on increase in airway epithelial mucosubstances from tracheal goblet cells of rats was assesed using histopathological analysis after staining the epithelial tissue with PAS-alcian blue. Possible cytotoxicity of MMT was assessed by investigating the potential damage of kidney and liver function by measuring serum GOT/GPT activities and serum BUN concentration of rats and body weight gain during the experiment.

## Results

(1) MMT did not only inhibit but also increased MUC5AC mucin production and expression levels of the MUC5AC gene from NCI-H292 cells. (2) MMT did not decrease the amount of intraepithelial mucosubstances in the trachea of rats. (3) MMT did not show renal or hepatic toxicity and did not affect body weight gain of rats during the experiment.

## Conclusion

MMT might normalize the production and gene expression of airway mucin observed in various respiratory diseases accompanied by yin-deficiency, without *in vivo* toxicity to liver and kidney functions after oral administration.

